# Exploring anatomical variations of mandibular premolars and their clinical management: a case series

**DOI:** 10.3389/fdmed.2026.1769755

**Published:** 2026-02-27

**Authors:** Bridhi Jalan, Srishti Grover, Janina Loren DSouza, Annapoorna Shenoy, Nisha Gawade

**Affiliations:** Department of Conservative Dentistry and Endodontics, Manipal College of Dental Sciences Mangalore, Manipal Academy of Higher Education, Manipal, India

**Keywords:** anatomic variations, cone beam computed tomography, mandibular first premolar, mandibular second premolar, Vertucci classification

## Abstract

Anatomical variations in root canals pose significant challenges and may affect the course of treatment. Mandibular premolars usually have a single canal with single root. However, this case series highlights three mandibular premolars with distinct canal configurations. The first case involved a mandibular second premolar with Sert and Bayirli Type IX anatomy, characterised by one canal trifurcating into three different canals at the apex. In the second case, the Vertucci Type V configuration is in the mandibular second premolar. In the third case, a mandibular first premolar showed a Vertucci Type IV configuration, which remained distinct throughout its course. Cone-beam computed tomography (CBCT) is a reliable diagnostic tool for identifying these intricate canal morphologies. The use of magnification ensured through biomechanical preparation and obturation, which contributes to the success of endodontic therapy.

## Introduction

The aim of endodontic treatment is to completely debride the root canal, eliminating bacteria and noxious stimuli, and thereby providing a three–dimensional hermetic seal. However, the presence of root canal variations makes it difficult to achieve these objectives (1). Endodontic failure is multifactorial and has been associated with a combination of biological, anatomical, and technical factors. Biological factors include the persistence of intraradicular or extraradicular infection, biofilm formation, untreated or missed canals, and coronal microleakage, which can lead to reinfection. Anatomical factors, particularly variations in root canal morphology such as additional canals, complex canal configurations, lateral and accessory canals, apical deltas, isthmuses, and canal curvatures, pose significant challenges to effective cleaning, shaping, and disinfection. Technical factors, including inadequate access cavity design, insufficient cleaning and shaping, iatrogenic procedural errors, suboptimal irrigation protocols, and deficiencies in obturation or coronal sealing, may further compromise treatment outcomes when these anatomical complexities are not adequately recognised and managed (2–4). Therefore, a thorough understanding of root canal anatomy and morphological variations, combined with meticulous execution of clinical procedures, is essential for achieving predictable success in endodontics.

Mandibular premolars(M_d_PM) are traditionally described as “single-rooted” teeth with a “single” root canal; however, studies show that approximately 24% of these teeth have two or more root canals (5). A systematic review reported that mandibular first premolars are more likely to exhibit canal bifurcation (23%–30%), terminating in numerous apical foramina (15%–20%), compared to second premolars (6).

The M_d_PM1 and M_d_PM2 root canal morphologies significantly differed among the South Indian population. According to Vertucci's classification, MdPM1 most commonly exhibited Type I (83.81%) and Type IV (10%) canal configurations, with lower frequencies of Type II (6%), Type V (2%), and Type IX (2%). In contrast, MdPM2 demonstrated a reduced prevalence of Type I anatomy (66%) and a markedly higher occurrence of Type II configurations (30%), with Type V observed in 4% of cases and no other canal patterns identified (2). Additionally, previous literature has reported the prevalence of Type IX canal configuration in MdPM2 to be exceedingly rare (0.4%) (7).

Several authors have proposed classification systems to describe root canal anatomy. Weine et al. (8) were the first to introduce a clinical classification for teeth with a single root. Subsequently, Vertucci (9) developed a comprehensive classification, which, along with its later modifications by Sert and Bayirli (10), expanded the system by introducing fourteen additional canal types, designated as Types IX–XXIII, and remains the most widely used system in endodontic literature. Gulabivala and colleagues (11) further contributed by reporting seven additional canal configurations.

The present case series we will discusses the various challegenes faced in identifying and treating discusses the various methods in identifying variations in root canal anatomy and the challenges encountered during treatment.

## Case reports

This case series adhered to the Preferred Reporting Items for Case Reports in Endodontics (PRICE) 2020 Guidelines (12). A comprehensive history was taken, along with thorough clinical and radiographic examinations. The medical histories of all three patients were non-contributory. The pulp sensibility test was performed using (Roeko Endo-Frost, Coltene, Europe) for the cold test; for the electric pulp test (Electric Pulp Tester, Waldent, New Delhi) was used, and for the heat test, a hot gutta-percha (Dentsply Maillefer, Ballaigues, Switzerland) was used.

After a detailed explanation of the case specifics and limitations, the patient provided informed consent, agreeing to proceed with the proposed endodontic treatment.

### Case 1

**Figure 1:**
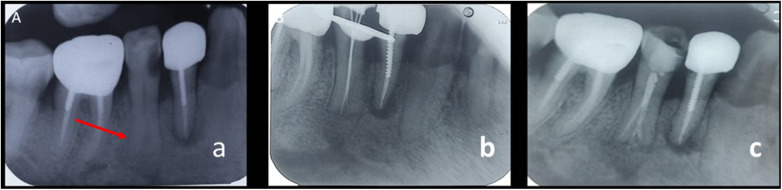
**(a)** preoperative IOPAR depicting root bifurcation with respect to 35 **(b)** working length IOPAR depicting Sert and Bayerli classification. IX. **(c)** Post-obturation IOPAR.

**Figure 2:**
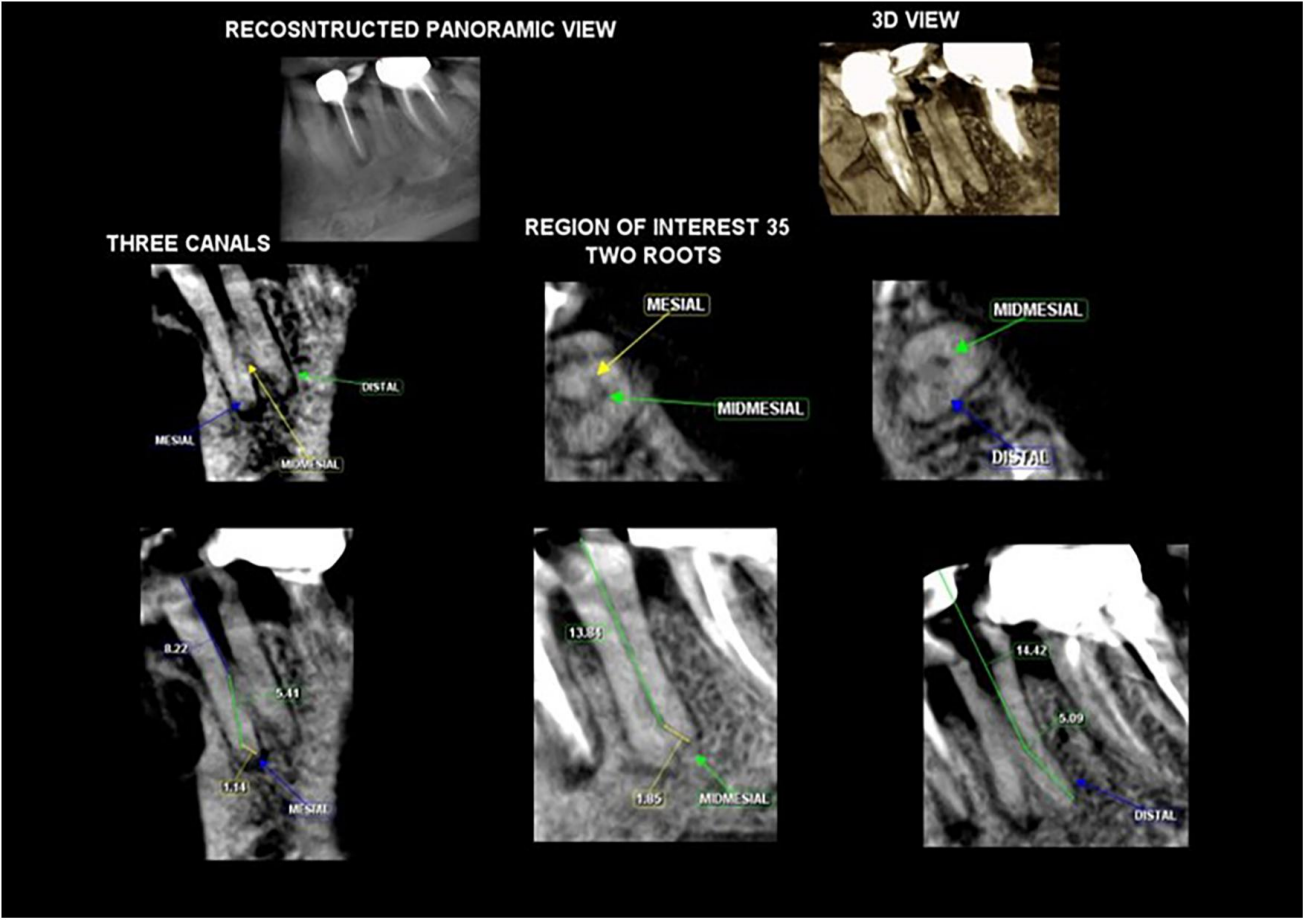
CBCT confirming the presence of 2 roots and 3 canals.

A 52-year-old Dravidian patient reported a complaint of pain in the lower left back tooth for the past 10 days. On eliciting history, it was noted that the patient experienced persistent pain for a week, which did not subside despite taking medication over the last three days. The pain was aching, continuous and radiating in nature. On clinical examination, tenderness on percussion was positive with respect to 35. Pulp sensibility tests revealed a lingering response to cold, heat and electric pulp. Radiographic examination revealed radiolucency extending through the enamel and dentin, approaching the pulp, with no peri-apical changes. Additionally, a bifurcated root was observed in the apical area, as depicted in (Figure 1a). A diagnosis of symptomatic irreversible pulpitis with symptomatic apical periodontitis was established based on clinical and radiographic findings, and root canal treatment was recommended. To gain a clearer understanding of the root canal anatomy, a CBCT scan was advised. The CBCT image showed a single canal with two roots, which trifurcated at the apical third, exiting as three separate canals, corresponding to Vertucci Type IX configuration (Figure 2).

The inferior alveolar nerve block with 2% lignocaine containing 1:200,000 epinephrine (xylocaine, AstraZeneca Pharma Ind Ltd., Bengaluru, India) was given. The procedure was performed under rubber dam isolation and a dental operating microscope (DOM) (Opmi Pico, Carl Zeiss, Oberkochen, Germany). Access opening was done, a glide path was established, with a 10 K file (Mani Inc., Japan), and the working length was determined using an apex locator (Coltene Canalpro CL2i, Coltene Whaledent, Inc., USA) and confirmed radiographically (Figure 1b). However, distinguishing between the Mesial Buccal, Mid-Mesial(lingual) and Distal Buccal was challenging on clinical inspection. The three canal orifices were viewed under 16x magnification in DOM. ProTaper SX (Dentsply Maillefer, Ballaigues, Switzerland) was used to perform coronal flaring using the crown-down technique. All three canals were subsequently prepared until 20 K file (Mani Inc., Japan). Final apical preparation was completed up to 25.04% using a rotary file (Neoendo S-S shaped cross-section Rotary Files, Orikam Healthcare India Pvt. Ltd.) along with irrigation using 3% sodium hypochlorite and normal saline. The irrigant was ultrasonically agitated using Acteon IrriSafe (K25/21 mm) for 20 s intervals, three times, to enhance the effectiveness of sodium hypochlorite.

In the subsequent visit, the canals were thoroughly irrigated with 17% ethylenediaminetetraacetic acid (EDTA) and normal saline. All canals were then dried using absorbent paper points and obturated with gutta-percha and AH Plus sealer (Dentsply Tulsa) (Figure 1c). The continuous wave of compaction technique was employed, followed by backfilling the canal with thermoplasticized gutta-percha.

### Case 2

**Figure 3:**
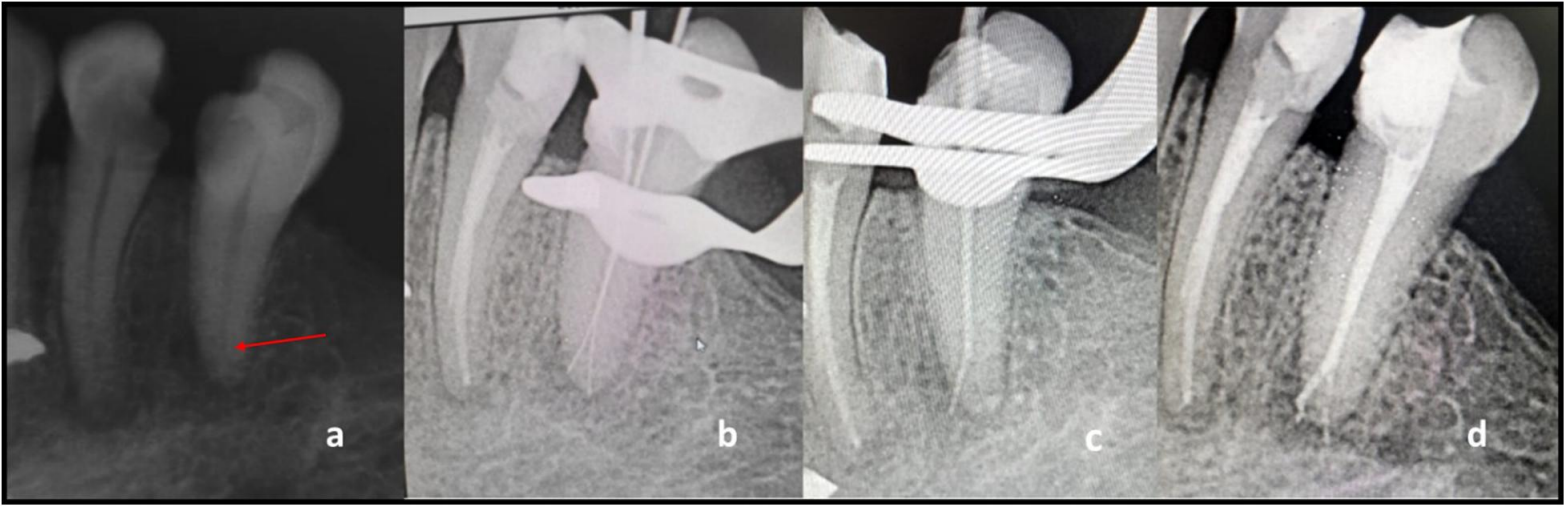
**(a)** Preoperative IOPAR) with respect to tooth 35 demonstrating the fast break principle; **(b)** Working length IOPAR depicting vertucci type V canal configuration; **(c)** Master cone IOPAR; **(d)** Post-obturation IOPAR.

A 35-year-old Dravidian patient presented with a complaint of pain in the lower left back tooth region for the past 7 days. The pain was aggravated during food lodgement and relieved when the lodged food particle was removed. Clinical examination revealed tenderness on vertical percussion, and a lingering response was noted during pulp sensibility testing. Upon radiographic examination, coronal radiolucency approximating pulp was noted along with the widening of the periodontal ligament space. The possibility of an extra canal was suspected as the canal outline faded towards the apical third, as depicted in (Figure 3a). Non-surgical endodontic treatment was chosen as the best treatment option after the patient was diagnosed with symptomatic irreversible pulpitis accompanied by symptomatic apical periodontitis.

The inferior alveolar nerve block was administered, and the procedure was performed under rubber dam isolation. DOM was used during access opening, and a glide path was established via a 10 K file. The working length was determined with an apex locator (Coltene Canalpro CL2i, Coltene Whaledent, Inc., USA), and radiographically (Figure 3b). Vertucci Type V canal configuration was confirmed. Chemico-mechanical preparation was performed via a crown-down technique with rotary files (Neoendo S- S-Shaped Cross-section Rotary Files, Orikam Healthcare India Pvt. Ltd.) till apical size 25.04% for both the canals. 3% sodium hypochlorite and normal saline were used alternately, and sodium hypochlorite was ultrasonically activated using Acteon® IrriSafe™ (K25/21 mm) for 20 s intervals three times. The canals were thoroughly irrigated with 17% EDTA and normal saline in the next visit. All canals were dried with absorbent paper points, and master cones were selected (Figure 3c). Obturation was done using Bioroot RC Sealer (Septodont, Saint-Maur-des-Fossés, France) and gutta-percha following lateral compaction technique (Figure 3d).

### Case 3

**Figure 4:**
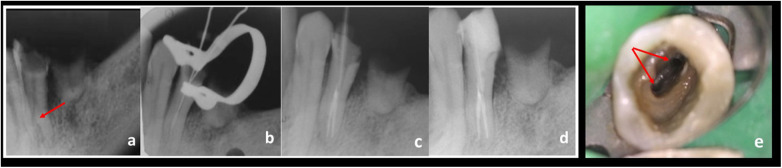
**(a)** Preoperative IOPAR depicting split roots with respect to 34. **(b)** Working length IOPAR depicting vertucci type IV classification. **(c)** Fibre post IOPAR. **(d)** Post-Obturation IOPAR. **(e)** Access opening showing 2 canal orifices under a dental operating microscope (DOM).

A 40-year-old Dravidian patient complained of pain in the left lower back tooth. Pain was intermittent and aggravated by drinking cold water; it lingered and subsided once the stimulus was removed. On clinical examination, deep dental caries were observed with 34, and the tooth was non-tender. Pulp sensitivity tests revealed lingering responses to cold and delayed responses to heat and to EPT. Radiographic radiolucency was observed in the enamel, dentin, and pulp, with no periapical change. The canal was seen bifurcating at the middle third, with root splitting into two, as depicted; this case was classified as Vertucci Type IV. The diagnosis was symptomatic irreversible pulpitis with asymptomatic apical periodontitis. The treatment plan chosen was nonsurgical root canal treatment with fibre post and core. Endodontic treatment was conducted as described in the case 2 mentioned above (Figures 4b–e).

## Discussion

The bifurcation of the root canal occurs due to the epithelial root sheath (HERS), which plays a key role in the development of the root and root canal system. A single collar-shaped diaphragm that develops into a single-rooted tooth or several tongue-like extensions that develop into several rooted teeth are the two ways in which HERS extends (13).

Before beginning root canal therapy, obtaining preoperative for diagnosis and intraoperative radiographs from various angles is essential to detect additional roots or canals. Diagnosis was done in accordance with the American Association of Endodontists (AAE) Consensus Conference–recommended diagnostic terminology. Each diagnostic category is defined based on its characteristic clinical findings and corresponding radiographic features (14). A phenomenon known as the “fast break principle” describes the sudden disappearance of a canal on a radiograph, which typically occurs because smaller, invisible canals branch out (2). Following the ALARA principle, which aims to reduce radiation exposure, The American Association of Endodontists recommends exercising caution when using CBCT imaging. A limited field-of-view (FOV) CBCT should be the imaging modality of choice for preoperative diagnosis in patients presenting with contradictory or nonspecific clinical symptoms and/or radiographic findings (15). CBCT should be used only when standard radiographs are insufficient. A targeted CBCT scan slightly larger than the area of interest is advised to lower radiation and improve image quality. CBCT provides detailed information on tooth morphology, the number and location of root canals, and particular characteristics such as calcification and curvature (16).

Another challenge clinicians often face is visualising `canal openings and pinpointing bifurcation locations. Using a dental operating microscope will improve visualisation and help locate additional canals (17). The use of optical loupes for magnification and fiber optic illumination for extra lighting is helpful.

The biomechanical preparation of teeth with aberrant canal morphology is demanding. The use of preventive methods, such as the utilisation of fresh instruments, regularly inspecting for distortion in files, employing flexible Ni‒Ti rotary and hand files, and pre-curving hand files, should be encouraged for shaping the canal (18). Instruments with increased tapers should be avoided, as they can cause or increase the incidence of strip perforation (19). Initially, in all 3 cases described in this case report, manual instrumentation was performed using smaller-diameter hand files. The axial CBCT scans confirmed the mid-root separation of all three canals in the 1st case, which was a defining feature of the root canal system in such cases. M_d_PM with three root canals are distinguished by a pulp chamber shaped like a triangle and has a significant separation between the Mesial Buccal and the Distal buccal orifices (20). Currently, access cavity alteration and orifice confirmation are performed using this canal detection technique and keeping geometric configuration in mind while locating the canals. Three detected canals were manually prepared with K files, ranging from size #6 K files to #20 K. Finally, a rotary system was used, and canals were prepared until #25.04. For the 2nd and 3rd cases, the lingual canal was manually recreated after the major canal wall was tactilely examined. The opening of the lingual canal was identified via a pre-curved scouting 8 K-file. Then, the buccal and lingual canals were prepared to a smaller apical size and taper (#25/.04). Sufficient coronal canal flaring was carried out to expose the canal orifices in all three patients. Since there was not enough coronal space to hold multiple master cones simultaneously, a smaller K file was inserted into the bifurcated canal to block it, and simultaneously, the main canal was filled, and the GP was cut at the bifurcation. Similarly, all the canals were obturated, and the coronal section of the canal was backfilled with thermoplastized gutta percha for a hermitic seal (21).

Finally, it is imperative to emphasize that the actions described in this case series were critical for its efficient management. However, patience, persistence, and clinical knowledge are equally important to achieve this goal.

## Conclusion

Owing to their intricate canal networks, M_d_PM are sometimes regarded as the hardest teeth to successfully treat with endodontic therapy. Nonetheless, a number of advancements in diagnostics, magnification, surgical tools, and methods, and a current understanding of the anatomy of M_d_PM, undoubtedly increase the success rates of even the most difficult endodontic cases.

## Clinical relevance

These case series offer insights that promote greater awareness and reduce the risk of missing variations in root canal morphology. Discusses the clinical management of commonly encountered Type V and Type IV canal configurations, as well as the uncommon Type IX pattern. It provides valuable insights that enhance clinician awareness and reduce the likelihood of overlooking anatomical variations in root canal morphology, thereby facilitating accurate and efficient treatment. This case series highlights the pivotal role of advanced diagnostic aids, particularly cone-beam computed tomography (CBCT), along with meticulous clinical and preoperative radiographic evaluation, in the identification and management of the Type IX canal configuration in the mandibular second premolar. Furthermore, it highlights the importance of clinician knowledge, vigilance, and anticipation of additional canals to minimise the risk of missed canals.

## Limitation

The findings of this case series are inherently limited by the small patient group, which restricts generalisation of the observations to a broader population.

## Data Availability

The raw data supporting the conclusions of this article will be made available by the authors, without undue reservation.
